# Transport of estradiol-17β-glucuronide, estrone-3-sulfate and taurocholate across the endoplasmic reticulum membrane: evidence for different transport systems^☆^

**DOI:** 10.1016/j.bcp.2013.12.026

**Published:** 2014-03-01

**Authors:** Katrin Wlcek, Lia Hofstetter, Bruno Stieger

**Affiliations:** Department of Clinical Pharmacology and Toxicology, University Hospital Zurich, Rämistrasse 100, 8091 Zurich, Switzerland

**Keywords:** ABC, ATP binding cassette, CYPs, cytochrome P450s, DHEAS, dehydroepiandrosteronesulfate, ER, endoplasmic reticulum, E17βG, estradiol-17β-glucuronide, E3S, estrone-3-sulfate, G6P, glucose-6-phosphate, HPTLC, high performance thin layer chromatography, MRP2, multidrug resistance associated protein 2, NE, nuclear envelope, Ntcp, Na-taurocholate co-transporting polypeptide, Oatp, organic anion transporting polypeptide, RER, rough endoplasmic reticulum, SER, smooth endoplasmic reticulum, STS, steroidsulfatase, TC, taurocholate, UDPGA, Uridine diphosphate glucuronic acid, UGTs, Uridine diphosphate glucuronosyltransferases, Liver, Biotransformation, Endoplasmic reticulum, Estrone-3-sulfate, Estradiol-17β-glucuronide, Transport

## Abstract

Important reactions of drug metabolism, including UGT mediated glucuronidation and steroidsulfatase mediated hydrolysis of sulfates, take place in the endoplasmic reticulum (ER) of hepatocytes. Consequently, UGT generated glucuronides, like estradiol-17β-glucuronide, have to be translocated back into the cytoplasm to reach their site of excretion. Also steroidsulfatase substrates, including estrone-3-sulfate, have to cross the ER membrane to reach their site of hydrolysis. Based on their physicochemical properties such compounds are not favored for passive diffusion and therefore likely necessitate transport system(s) to cross the ER membrane in either direction.

The current study aims to investigate the transport of taurocholate, estradiol-17β-glucuronide, and estrone-3-sulfate in smooth (SER) and rough (RER) endoplasmic reticulum membrane vesicles isolated from Wistar and TR^−^ rat liver.

Time-dependent and bidirectional transport was demonstrated for taurocholate, showing higher uptake rates in SER than RER vesicles. For estradiol-17β-glucuronide a fast time-dependent efflux with similar efficiencies from SER and RER but no clear protein-mediated uptake was shown, indicating an asymmetric transport system for this substrate. Estrone-3-sulfate uptake was time-dependent and higher in SER than in RER vesicles. Inhibition of steroidsulfatase mediated estrone-3-sulfate hydrolysis decreased estrone-3-sulfate uptake but had no effect on taurocholate or estradiol-17β-glucuronide transport.

Based on inhibition studies and transport characteristics, three different transport mechanisms are suggested to be involved in the transport of taurocholate, estrone-3-sulfate and estradiol-17β-glucuronide across the ER membrane.

## Graphical abstract

**Figure:**
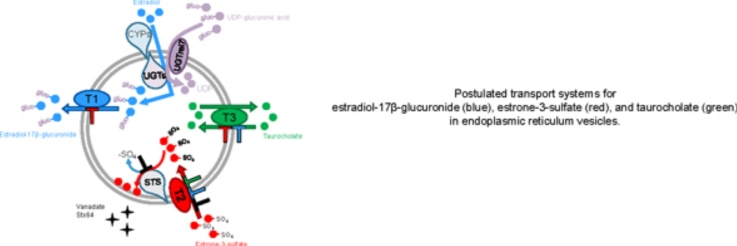

## 1 Introduction

Biotransformation and elimination of endogenous and exogenous compounds is an important function of the liver. The hepatic drug elimination process occurs in hepatocytes and is classified into four phases including drug uptake (phase 0), phase 1 and 2 biotransformation and excretion of metabolites (phase 3) [1]. Drug uptake and metabolite excretion is accomplished by transport proteins localized at the basolateral and canalicular membrane of hepatocytes. The transport systems localized to the plasma membrane are complemented by intracellular enzymes catalyzing phase 1 and phase 2 reactions, converting poorly water-soluble compounds into more water-soluble metabolites to enable their excretion into the bile or the sinusoidal blood plasma. Phase 1 reactions are mainly accomplished by members of the cytochrome P450 enzyme family (CYPs) preparing compounds for subsequent phase 2 conjugation. A major phase 2 pathway represents the conjugation with glucuronic acid mediated by the family of the UDP-glucuronosyltransferases (UGTs) [2]. In humans, CYPs and UGTs are involved in the clearance of 90% of drugs which undergo hepatic elimination but also in the conjugation of endogenous compounds, including estradiol or bilirubin [3]. Furthermore, glucuronidation can represent an alternative detoxification pathway for bile acid elimination in cholestasis to protect hepatocytes from the cytotoxic effect of bile acids in case of accumulation [4]. Elimination of glucuronides into bile is accomplished by the multidrug resistance-associated protein 2 (MRP2), which belongs to the ATP binding cassette (ABC) transporter superfamily [5]. The lack of functional MRP2 is known to be responsible for deficient glucuronide excretion into bile, resulting in glucuronide accumulation and conjugated hyperbilirubinemia, known as Dubin Johnson syndrome in humans [6]. A corresponding animal model are the so-called TR^−^ rats [7].

Both CYPs [8] as well as UGTs [9] are anchored in the membrane of the endoplasmic reticulum (ER). In addition, steroidsulfatase (STS, also known as estronesulfatase or arylsulfatase C), which is important for steroid activation, is anchored as another metabolizing enzyme in the ER membrane [10–12]. The ER is a heterogenous compartment structurally divided into three subcompartments, including the nuclear envelope (NE), the smooth (SER) and the rough (RER) endoplasmic reticulum [13]. The RER harbours membrane bound ribosomes [14] and therefore is involved in protein biosynthesis [15]. By contrast, the CYPs [8] and UGTs [16] are localized in the SER. Besides in SER, UGTs have also been detected in RER and the NE [16]. Localization of STS has been identified in the ER and the NE in rat liver [17], but its distribution between SER and RER is still unclear. Besides the shared localization of CYPs, UGTs, and STS in the ER membrane, these phase 1 and phase 2 enzymes differ in the localization of their catalytic sites with respect to the cytoplasm. While the CYPs have their catalytic site in the cytoplasm [18], the active sites of UGTs [19] and STS [12] are located in the lumen of the ER. Consequently, substrates of STS, like estrone-3-sulfate (E3S) or dehydroepiandrosteronesulfate (DHEAS), have to cross the ER membrane to reach their site of hydrolysis. Due to their physicochemical properties, these compounds are not favored for passive diffusion and very likely necessitate transport system(s) to get access to the ER lumen. However, until now, no data are available on the characterization of the transport of the STS model substrate estrone-3-sulfate across the ER membrane.

Accordingly, the co-substrate for UGT mediated glucuronidation, the uridine diphosphate glucuronic acid (UDPGA) has to be translocated from its site of synthesis in the cytoplasm, to its site of action within the ER lumen. After UGT mediated glucuronidation, the resulting products have to be transported from the ER lumen back into the cytoplasm. Based on their physicochemical properties neither UDPGA nor glucuronides are likely to cross the ER membrane by simple diffusion, indicating the involvement of transport system(s) in the translocation of these compounds across the ER membrane. Evidence for the existence of such transport system(s) is given as UDPGA transport has been reported in rat liver microsomes [20]. Transport activity into microsomes isolated from rat liver has also been shown for different glucuronides [21–24]. However, only one study investigated transport of estradiol-17β-glucuronide in ER enriched liver microsomes but this study did not distinguish between SER and RER. Finally, carrier-mediated transport of taurocholate (TC) into rat liver microsomes, largely being composed of ER has been reported a while ago [25].

Based on the knowledge that STS and UGTs have their catalytic site within the ER lumen, the present study investigated and characterized the transport activity for the UGT model product estradiol-17β-glucuronide (E17βG) as well as the STS model substrate estrone-3-sulfate (E3S) in SER and RER vesicles isolated from rat liver. Transport of sulfated and glucuronidated model compounds across SER and RER membranes were functionally compared. Furthermore, a potential impact of a chronic cytoplasmic accumulation of glucuronides on the activity of these transport systems was investigated using ER fractions isolated from wild type and from the TR^−^ rats. Finally, taurocholate transport into RER and SER was investigated to test for potential common transport pathways with phase 2 products.

## 2 Materials and Methods

### 2.1 Materials

[6,7-^3^H(N)]-estradiol-17β-glucuronide (E17βG, 41.8 Ci/mmol), [6,7-^3^H(N)]-estrone-3-sulfate (E3S, 45.6 Ci/mmol), [^3^H(G)]-taurocholic acid (TC, 5 Ci/mmol) as well as the liquid scintillation fluid Filter Count were purchased from Perkin-Elmer (Schwerzenbach, Switzerland). Unlabelled substrates including E3S, E17βG, and TC as well as valinomycin, puromycin, and ATP were purchased from Sigma Aldrich (Buchs, Switzerland). Compounds used for inhibition studies, including UDP-glucuronic acid (UDPGA), Stx64, and vanadate were purchased from Sigma Aldrich (Buchs Switzerland). Glucose-6-phosphate was purchased from Roche (Basel, Switzerland) and bovine serum albumin was purchased from Interchim (Montluçon, France). All chemicals used were of the highest purity available. HPTLC plates and solvents used for HPTLC analysis were purchased from Merck (Darmstadt, Germany).

### 2.2 Animals

Wistar rats, obtained from Charles River (Charles River Laboratories, Sulzfeld, Germany), Sprague Dawley rats obtained from Harlan (Harlan Laboratories, Horst, Netherlands), and TR^−^ rats (180–200 g), were held in the animal facilities of the University Hospital of Zurich. The animals received humane care in accordance with local and federal guidelines. Animal experiments were approved by the local government's animal care committee.

### 2.3 Membrane vesicle isolation

#### 2.3.1 Isolation of basolateral and canalicular membrane vesicles from rat liver

Basolateral and canalicular membrane vesicles were isolated from Sprague Dawley rats as described by Meier and colleagues [26]. After isolation, vesicles were rapidly frozen and stored in liquid nitrogen until use and protein content was measured using the method of Lowry [27].

#### 2.3.2 Isolation of smooth (SER) and rough (RER) endoplasmic reticulum vesicles from rat liver

SER and RER vesicles were isolated from livers of overnight fasted male Wistar and TR^−^ rats (150-200 g) by density gradient centrifugation following procedures as described previously (procedure B, [28]). After removal of the livers from the animals all steps were performed on ice or in the cold room. After density gradient centrifugation the SER fraction, located at the 0.6 M and 1.3 M sucrose interface, was collected by careful aspiration. The pelleted RER was rehomogenized in 0.25 M sucrose in a glass Teflon homogenizer at full speed. Subsequently, SER and RER vesicles were recovered by centrifugation at 120 000 g for 45 min at 4 °C. The supernatant was discarded and pelleted SER and RER vesicles were resuspended in the desired resuspension buffer using a 25G syringe needle. Vesicles were rapidly frozen and stored in liquid nitrogen until use. Protein of SER and RER vesicles was determined using bicinchoninic acid (Thermo Scientific, Waltham, Massachusetts) and bovine serum albumin as standard.

### 2.4 Western blotting

Proteins were separated by SDS-PAGE using 7.5% separating gels followed by blotting on nitrocellulose membranes (GE Healthcare, Little Chalfont, United Kingdom) with standard procedures. Membrane fractions were probed for Cyp3a2 (Enzo Lifesciences CR3320, Lausen, Switzerland; 1:7000 dilution), Sec61α (Thermo Scientific PA3-014, Waltham, Massachusetts; dilution 1:7000) or PDI (Thermo scientific MA3-019, Waltham, Massachusetts; dilution 1:20 000 or 1:100 000) by either incubation at 4 °C overnight (Sec61α) or at room temperature for 2 h (Cyp3a2; PDI). Either HRP-conjugated goat anti rabbit IgG (GE Healthcare RPN4301, Little Chalfont, United Kingdom; for Sec61α and Cyp3a2; dilution 1:30 000) or sheep anti mouse IgG (GE Healthcare NA931VS, Little Chalfont, United Kingdom; dilution 1:3000) was used as secondary antibody. All antibodies were diluted in TBS-T (150 mM NaCl, 10 mM Tris/HCl pH 7.6, 0.1% Tween 20) containing 5% milk powder. UptiLight™ (Interchim, Montluçon, France) was used as chemiluminescent substrate for protein detection. Western blot analyses for Cyp3a2 and Sec61α were performed for each SER and RER isolation used in this study.

Primary antibodies against Oatp1a1 [29], Ntcp [30], and Mrp2 [31] were used in 1:1000, 1:5000, and 1:2000 dilutions in 5% milk/TBS-T, respectively. For stripping off antibodies, nitrocellulose membranes were incubated for 30 min at room temperature in stripping buffer (100 mM NaCl, 100 mM glycine, pH adjusted to 3.0 with HCl) followed by three washing steps in TBS-T for 10 min. Incubation with secondary HRP-conjugated goat anti rabbit IgG (GE Healthcare RPN4301, Little Chalfont, United Kingdom) and protein detection with UptiLight™ (Interchim, Montluçon, France) was performed as described above.

### 2.5 Transport studies

#### 2.5.1 Vesicle uptake studies

Uptake of radioactive labelled substrates was measured by the rapid filtration method [32]. Vesicles were quickly thawed in a 37 °C waterbath, diluted to a protein concentration of 3.5 mg/ml with the appropriate resuspension buffer and revesiculated by 20 time aspiration through a 25G needle with a syringe. 20 μl (70 μg) of vesicles were prewarmed at 37 °C for 30 sec and uptake was initiated by the addition of 80 μl of incubation buffer containing the tritiated substrate. After the desired time of incubation transport was stopped by the addition of 3 ml ice-cold stop solution (50 mM sucrose, 100 mM KCl, 0.1 mM Ca(gluconate)_2_, 5 mM MgCl_2_ and 20 mM Hepes/Tris pH 7.4) and solution was rapidly filtered through a 0.45 μm nitrocellulose acetate filter (Sartorius, Göttingen, Germany). For TC transport studies, filters were presoaked with 3 ml of 1 mM TC solution. Filters were dissolved in Filter Count (Perkin Elmer, Schwerzenbach, Switzerland) and radioactivity was measured by liquid scintillation counting in a Betacounter (Perkin Elmer, Schwerzenbach, Switzerland).

##### 2.5.1.1 Transport studies at different membrane potentials

To test for the role of the membrane potential on transport activities, vesicles were pretreated with valinomycin (10 μg valinomycin per mg protein), added from a stock solution (10 mg/ml in DMSO), for 30 min at room temperature followed by 30 min incubation on ice prior to transport studies. Subsequently a membrane potential was generated by either an inward or outward directed potassium gradient. To generate an inward positive membrane potential, vesicles were resuspended in 250 mM sucrose, 0.1 mM Ca(gluconate)_2_, 20 mM Hepes/Tris, pH 7.4 (K_out_ > K_in_ resuspension buffer) and substrate containing incubation buffer (K_out_ > K_in_ incubation buffer) consisted of 50 mM sucrose, 100 mM Kgluconate, 0.1 mM Ca(gluconate)_2_, 2.5 mM Mg(gluconate)_2_, 20 mM Hepes/Tris pH 7.4. Contrary, an inside negative membrane potential was generated by using 50 mM sucrose, 100 mM Kgluconate, 0.1 mM Ca(gluconate)_2_, 20 mM Hepes/Tris pH 7.4 as resuspension buffer (K_out_ < K_in_ resuspension buffer) and 250 mM sucrose, 0.1 mM Ca(gluconate)_2_, 2.5 mM Mg(gluconate)_2_, 20 mM Hepes/Tris, pH 7.4 as incubation buffer (K_out_ < K_in_ incubation buffer).

##### 2.5.1.2 ATP dependent uptake studies

For ATP dependent uptake studies, vesicles were resuspended in 50 mM sucrose, 100 mM KNO_3_, 20 mM Hepes/Tris pH 7.4. The substrate containing incubation buffer either consisted of 50 mM sucrose, 100 mM KNO_3_, 12.5 mM Mg(NO_3_)_2_, 10 mM Hepes/Tris, pH 7.4 alone or with 6.25 mM ATP. Uptake was performed as described in 2.5.1.

##### 2.5.1.3 Detaching of ribosomes from RER vesicles

Attached ribosomes of RER vesicles were removed using puromycin and high KCl concentration as described previously [33]. SER vesicles were treated in parallel as control. Briefly, vesicles were treated in K_out_ > K_in_ resuspension buffer as described in 2.5.1.1. or 0.25 M sucrose, 0.75 M KCl, 1 mM puromycin, 50 mM Tris/HCl pH 7.4 for 1 h at room temperature. After centrifugation (15 min, 103 856 g) SER and ribosome-free RER vesicles were resuspended in K_out_ > K_in_ resuspension buffer. Protein of SER and RER vesicles was determined using bicinchoninic acid (Thermo Scientific, Waltham, Massachusetts) and bovine serum albumin as standard and uptake of TC was measured at an inward positive membrane potential as described in section 2.5.1 and 2.5.1.1.

##### 2.5.1.4 Na^+^-dependent taurocholate uptake

For sodium dependent TC transport, vesicles were resuspended in 250 mM sucrose, 0.1 mM Ca(gluconate)_2_, 20 mM Hepes/Tris, pH 7.4. The substrate containing incubation buffer consisted of 50 mM sucrose, 0.1 mM Ca(gluconate)_2_, 2.5 mM Mg(gluconate)_2_, 20 mM Hepes/Tris pH 7.4 and either 100 mM NaCl or KCl. Uptake studies were performed as described in section 2.5.1.

##### 2.5.1.5 Osmolarity plots

Experiments for generating osmolarity plots were performed at an inward positive membrane potential at an incubation time point of 10 min (see section 2.5.1.1.) [34].

Vesicles were resuspended in K_out_ > K_in_ resuspension buffer (see 2.5.1.1.) and the osmolarity outside of the vesicles was increased by adding increasing sucrose concentrations, ranging from 50 mM to 800 mM, to the K_out_ > K_in_ incubation buffer (see 2.5.1.1.). Uptake was performed as described in section 2.5.1.

Uptake (pmol/mg protein) was plotted against the reciprocal osmolarity of the incubation buffer and linear regression analyses were performed using the GraphPad prism software (GraphPad Software Inc., San Diego, USA). The amount of substrate binding to vesicles is given at the intercept of the extrapolated linear regression with the y-axis (uptake), whereas uptake rates observed at identical intra- and extravesicular osmolarities corresponding to 250 mM sucrose was taken as 100%.

#### 2.5.2 Vesicle efflux studies

For efflux studies, 100 μg vesicles were preincubated with the given concentration of radiolabelled substrate in resuspension buffer in parallel with valinomycin treatment (see section 2.5.1.1). Efflux was initiated by a 60- fold dilution with incubation buffer. After the desired time of incubation at 37 °C, efflux was stopped by the addition of ice-cold stop solution and vesicle associated radioactivity was measured as described in 2.5.1 Membrane potentials during efflux studies were generated as described before (section 2.5.1.1).

### 2.6 High performance thin layer chromatography (HPTLC) analysis

As described previously [22] 20 μl (3.5 mg/ml protein) vesicles were preincubated at 37 °C and mixed with 80 μl of E3S containing (5 μM, 3 μCi/ml) incubation solution to start the reaction. The mixture was incubated for the desired time period at 37 °C at a thermoshaker (900 rpm) and reaction was stopped by the addition of 100 μl ice cold ethanol. The samples were centrifuged at 20 200 g (14 000 rpm) for 10 min at 4 °C and 25 μl of the supernatant were loaded on HPTLC Silicagel 60 plates (Merck, Darmstadt, Germany) using an automated TLC sampler (Camag, ATS4, Muttenz, Switzerland). Chromatography was performed using a solvent mixture of ethyl acetate:methanol:25% ammoniumhydroxide solution at a volume ratio of 37.5:12.5:1. The developing chamber was saturated with the same solvent mixture from 10 min prior to development of the HPTLC plate. The HPTLC plate was developed until the solvent front reached 8 cm from the bottom of the plate (∼15 min). The plate was dried at 100 °C for 10 min and a X-ray film (Fujifilm, Dielsdorf, Switzerland) was exposed for 9 days to 1 month.

### 2.7 Statistical analysis

Data are given as means ± SD. The number of independent experiments is given in the figure and table legends.

Where indicated, statistical significance was calculated using unpaired t-test and the GraphPad prism software (GraphPad Software Inc., San Diego, USA). A p-value <0.05 was considered as statistical significant.

## 3 Results

### 3.1 Characterization of SER and RER vesicles

To test for successful separation of SER and RER, expression of proteins known to be present in different ER compartments (Cyp3a2, Sec61α and the protein disulfid isomerase PDI), was analyzed by Western blotting.

**Fig. 1:**
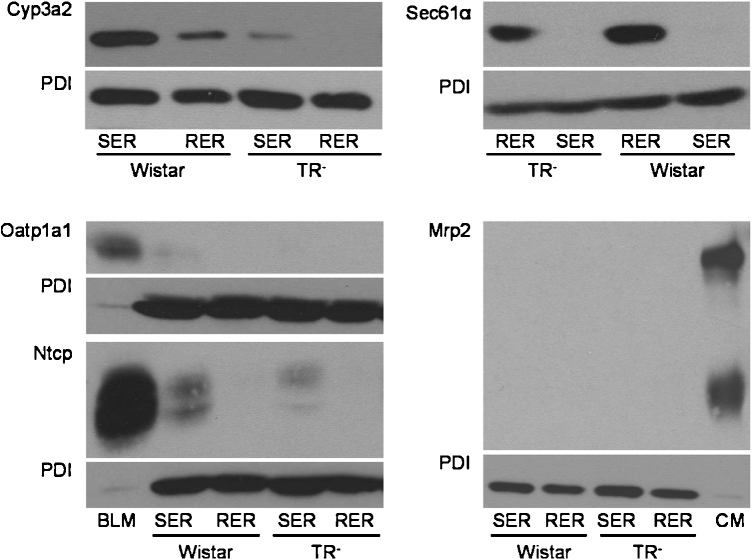
Western blot analysis of Cyp3a2, Sec61α, Oatp1a1, Ntcp, Mrp2, and PDI in SER and RER isolated from Wistar and TR^−^ rat livers. 5 μg (Cyp3a2, Mrp2), 50 μg (Sec61α) or 100 μg (Oatp1a1, Ntcp) protein was loaded per lane and separated by SDS-PAGE as given in section 2.4. Samples were probed against Cyp3a2, Sec61α, Oatp1a1, Ntcp, Mrp2, and PDI as described in 2.4. PDI was used as loading control in SER and RER fractions. Basolateral membrane fraction (BLM) isolated from Sprague Dawley rat liver was used as positive control for Oatp1a1 and Ntcp expression. Canalicular (CM) membrane fraction isolated from Sprague Dawley rat liver was used as positive control for Mrp2 expression. The figure shows a representative Western blot of at least two independent SER and RER isolations.

While PDI is reported to be equally distributed between SER and RER [35], Sec61α is enriched in the RER [35] and Cyp3a2 in the SER [8]. These markers were routinely assessed in the isolated SER and RER fractions. In our ER subfractions, PDI showed, as published, similar expression levels in SER and RER and was therefore chosen as loading control (Fig. 1). Sec61α expression was indeed restricted to the RER fractions and Cyp3a2 expression was detected at considerably higher levels in SER as compared to the RER fractions but did not reach statistical significance in densitometric measurements (Table 1). Hence, we successfully isolated membrane fractions enriched in RER or in SER. Interestingly, TR^−^ rats showed decreased Cyp3a2 expression compared to Wistar rats, which is in accordance to previous data [36], while Sec61α was expressed at similar levels in both rat strains (Fig. 1, Table 1).

To assess potential cross-contamination of isolated vesicles with plasma membranes, protein expression of basolateral and canalicular membrane proteins, including Ntcp, Oatp1a1 and Mrp2, was also tested by Western blotting. In comparison to basolateral or canalicular membrane fractions of Male Sprague Dawley rats, used as positive controls, expression of all three proteins was only faint. Therefore, contamination of RER and SER fractions with plasma membrane is only marginal (Fig. 1).

### 3.2 Taurocholate transport

#### 3.2.1 Taurocholate uptake

**Fig. 2:**
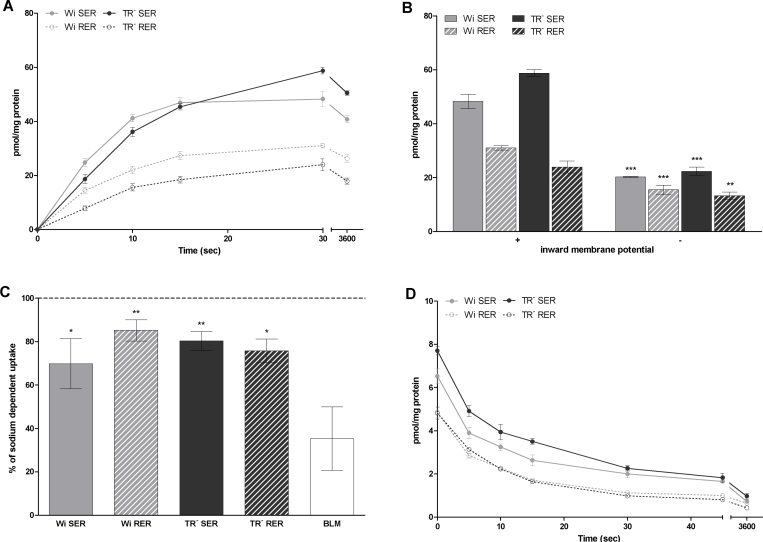
TC uptake (A, B, C) and efflux (D) in SER and RER of Wistar and TR^−^ rat liver. **A:** Uptake of 1 μM TC was determined in SER and RER fractions after different time points of incubation at 37 °C at an inward positive membrane potential as described in section 2.5.1. Data are shown for a representative sample of 3 independent experiments where uptake is calculated as mean pmol/mg protein ± SD from measurements at least in triplicates. **B:** Uptake of 1 μM TC was determined in SER and RER fractions after 30 sec of incubation at an inward positive (+) or negative (-) membrane potential as described in section 2.5.1.1. Data are shown for a representative sample of 3 independent experiments where uptake is calculated as mean pmol/mg protein ± SD from measurements at least in triplicates. Statistically significant difference in TC uptake between an inward positive and negative membrane potential are indicated by asterisks as followed: ** for p < 0.01 and *** for p < 0.001. **C:** Uptake of 1 μM TC was determined in SER, RER fractions and basolateral membrane (BLM) after 30 sec of incubation at 37 °C in presence and absence of sodium as described in section 2.5.1.4. Data are shown for a representative sample of 2 independent experiments. Data show TC uptake in absence of sodium given as % of uptake measured in presence of sodium. Values are given as means ± SD from measurements at least in triplicates. Statistically significant difference in TC uptake between ER fractions and basolateral membrane vesicles are indicated by asterisks as followed: * for p < 0.05 and ** for p < 0.01. **D:** Efflux of TC out of SER and RER vesicles preloaded with 1 μM TC was measured at an inward positive membrane at 30 sec of incubation at 37 °C as described in 2.5.2. Data (mean ± SD from measurements in triplicates) are shown for a representative sample of 2 independent experiments.

Uptake of taurocholate (TC) into SER and RER vesicles was time-dependent and electrogenic (Fig. 2A and 2B), showing 1.8 to 2.6 higher transport rates at an inward positive membrane potential compared to an inward negative membrane potential generated by potassium gradients and valinomycin treatment at 30 sec of incubation (Fig. 2B). Hence, the isolated SER and RER vesicles are transport competent and display electrogenic TC uptake, as previously demonstrated for rat liver microsomes [25]. Additionally, TC uptake showed an overshoot phenomenon, with an about 1.2 fold higher uptake at 30 sec compared of uptake rates observed after 1 h in SER and RER isolated from Wistar and TR^−^ rat liver (Fig. 2A).

Comparing TC uptake rates in SER and RER vesicles, TC uptake was about 1.5 fold higher in SER than in RER vesicles in Wistar rats at an incubation time of 30 sec. Also TR^−^ rats showed a 2.5 fold higher TC uptake at this time point of incubation into SER compared to RER vesicles (Fig. 2A). As RER vesicles can have a higher protein content due to the attached ribosomes than SER vesicles, SER and RER vesicles were treated with puromycin and KCl (see section 2.5.1.3.) to strip off attached ribosomes. Even after removal of ribosomes from RER vesicles, uptake of TC into SER vesicles was 2.7 fold higher than into RER vesicles (data not shown).

To exclude that the observed TC transport is due to contamination by basolateral membranes, sodium-dependent Ntcp-mediated TC uptake was measured (Fig. 2C). Basolateral membrane vesicles, which were used as positive control for Ntcp-mediated sodium-dependent TC transport, showed only 35% sodium independent of total TC transport. Contrary, 67-83% of total TC transport was sodium independent in SER and RER vesicles isolated from Wistar and TR^−^ rat livers. Consequently, only a marginal sodium-dependent (Ntcp-mediated) transport activity for TC was shown in SER and RER fractions compared to basolateral membrane fractions, supporting minimal expression of these proteins observed by Western blotting (Fig. 1) and consequently attributing the electrogenic TC transport activity to SER and RER.

Comparing vesicles isolated from Wistar and TR^−^ rats, both rat strains showed comparable uptake characteristics for TC (Fig. 2).

#### 3.2.2 Taurocholate efflux

As found for TC uptake, efflux of this compound out of SER and RER vesicles isolated from Wistar and TR^−^ rat liver was also time-dependent, indicating bidirectional transport of TC across the ER membrane (Fig. 2D). Within 30 sec of incubation about 70-80% of TC was transported out of SER and RER vesicles. Again, differences in TC efflux between Wistar and TR^−^ rats were only minimal.

#### 3.2.3 Taurocholate transport versus binding

**Fig. 3:**
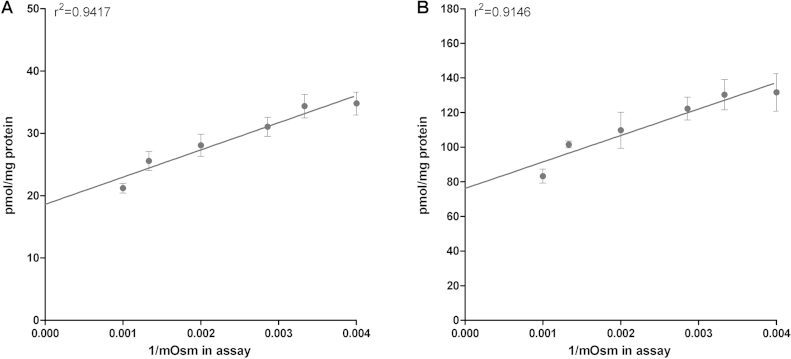
Osmolarity plot for TC (A) and E17βG (B) in SER of Wistar rat liver. Uptake of TC (1 μM) or E17βG (5 μM) was measured after an incubation time of 10 min at 37 °C at an inward positive membrane potential as described in 2.5.1.5. Outside osmolarity (Osm) was increased by adding sucrose ranging from 50 to 800 mM. Data (mean ± SD and linear regression analysis) are shown for a representative sample of 2 independent experiments.

To discriminate between intravesicular uptake and unspecific binding of the substrate to the vesicle membrane, osmolarity plots were performed. As shown in Fig. 3A, osmolarity plot for TC demonstrated a slope significantly different from zero. Consequently, active transport for TC can be assumed. However, 53% vesicle associated TC accounted for binding to membranes.

### 3.3 Estradiol-17β-glucuronide transport

#### 3.3.1 Estradiol-17β-glucuronide uptake and efflux

**Fig. 4:**
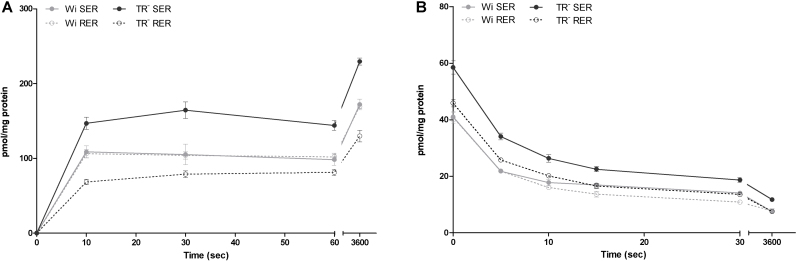
Time-dependent uptake (A) and efflux (B) of E17βG in SER and RER of Wistar and TR^−^ rat liver. **A:** Uptake of 5 μM E17βG was determined in SER and RER fractions after different time points of incubation at 37 °C at an inward positive membrane potential as described in 2.5.1. Data are shown for a representative sample of 3 independent experiments where uptake is calculated as mean pmol/mg protein ± SD from measurements at least in triplicates. **B:** Effux of E17βG out of E17βG (5 μM) preloaded vesicles was measured at an inward positive membrane potential after different time points at 37 °C as described in 2.5.2. Data (mean ± SD from measurements in triplicates) are shown for a representative sample of 2 independent experiments.

For E17βG a fast initial uptake within the first 10 sec of incubation was found (Fig. 4A). No change in uptake rates with increasing incubation time was seen between 10 sec and 1 min. Nevertheless, at equilibrium (after 1 h), E17βG uptake was 1.6 to 1.9 fold higher as compared to the uptake rates observed at an incubation time of 10 sec (Fig. 4A). The presence of ATP did not increase E17βG uptake rates (Table 2), indicating the absence of a functional Mrp in this membrane preparation and confirming data of Western blot analysis (Fig. 1).

#### 3.3.2 Estradiol-17β-glucuronide efflux

A fast efflux of E17βG could be detected in both microsomal fractions of Wistar and TR^−^ rat livers (Fig. 4B), showing export of 42-47% of E17βG already after 5 sec of incubation. Efflux of E17βG increased with time and after 30 sec 66-73% of preloaded E17βG were released. At 1 h of incubation only 16-20% of the substrates were left in the vesicles. Similar efflux rates were observed in SER and RER. Wistar and TR^−^ rats showed comparable characteristics of E17βG efflux.

#### 3.3.3 Estradiol-17β-glucuronide transport versus binding

Osmolarity plots for E17βG were performed to discriminate between intravesicular uptake and unspecific binding. A slope significantly different from zero was shown (Fig. 3B), demonstrating active transport for E17βG. However, 58% of vesicle associated E17βG account for membrane binding.

### 3.4 Estrone-3-sulfate transport

#### 3.4.1 Estrone-3-sulfate uptake

**Fig. 5:**
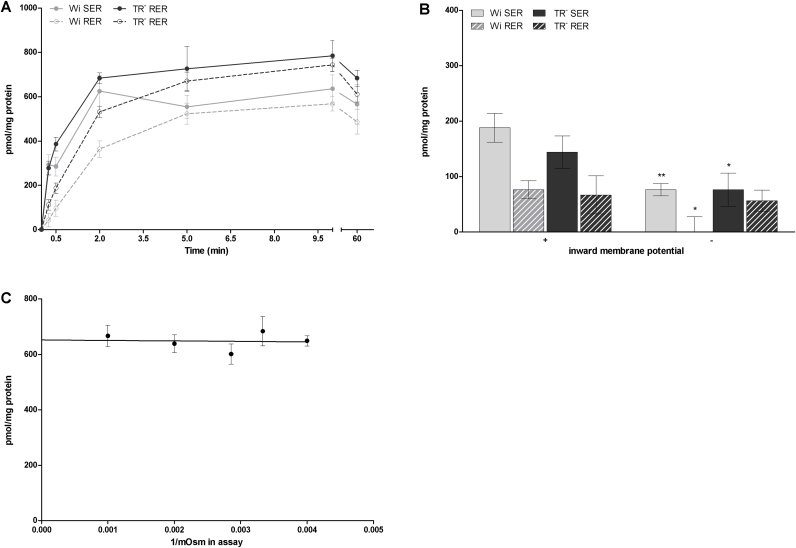
Uptake (A, B) and osmolarity blot (C) of E3S. **A:** Time-dependent uptake of 5 μM E3S was determined in SER and RER fractions of Wistar and TR^−^ rat liver after different time points of incubation at 37 °C at an inward positive membrane potential as described in 2.5.1. Uptake is calculated as mean pmol/mg protein ± SD from measurements at least in triplicates. Data are shown for a representative sample of 2 independent experiments. **B:** Uptake of 5 μM E3S was determined in SER and RER fractions of Wistar and TR^−^ rat liver after 10 sec of incubation at an inward positive (+) or negative (-) membrane potential as described in section 2.5.1.1. Data are shown for a representative sample of 2 independent experiments where uptake is calculated as mean pmol/mg protein ± SD from measurements in triplicates. Statistically significant difference in TC uptake between an inward positive and negative membrane potential are indicated by asterisks as followed: * for p < 0.05 and ** for p < 0.01. **C:** Uptake of E3S (5 μM) was measured in SER of Wistar rat liver after an incubation time of 10 min at 37 °C at an inward positive membrane potential as described in 2.5.1.5. Outside osmolarity (Osm) was increased by adding sucrose ranging from 50 to 800 mM. Data (mean ± SD and linear regression analysis) are shown for a representative sample of 2 independent experiments.

The transport of estrone-3-sulfate (E3S) in SER and RER vesicles isolated from Wistar and TR^−^ rat liver was time-dependent. A fast increase of E3S uptake was detected within the first 2 min of incubation in SER and RER vesicles, (Fig. 5A) remaining nearly constant between 5 and 10 min of incubation. At equilibrium after 1 h of incubation E3S uptake rates slightly decreased to 82-89% of levels observed at 10 min of incubation (Fig. 5A). Higher E3S uptake rates were detected at an inward positive membrane potential compared to an inward negative membrane potential, showing a 2.5- and 1.9 fold increase in SER membrane vesicles isolated from Wistar and TR^−^ rat livers, respectively after 10 sec of incubation (Fig. 5B). Consequently, an inwardly directed positive membrane potential was chosen for further transport experiments.

As observed for TC, E3S showed higher uptake rates in SER compared to RER membrane fractions. In Wistar rats, E3S uptake was 6.8 fold higher in SER than in RER at 15 sec of incubation (Fig. 5A). Likewise, in SER isolated from TR^−^ rats a 2.5 fold increase of E3S uptake compared to the RER fraction was observed (Fig. 5A).

Similar to TC transport, Wistar and TR^−^ rats showed comparable transport characteristics for E3S.

#### 3.4.2 Estrone-3-sulfate transport versus binding

In contrast to TC and E17βG, E3S osmolarity plots showed no transport into an osmotically active space, suggesting binding of the substrate to or partioning into the vesicle membranes (Fig. 5C).

#### 3.4.3 Role of steroidsulfatase in estrone-3-sulfate transport

As E3S uptake into SER and RER vesicles was time-dependent, but showed 100% binding to vesicle membranes, high performance thin layer chromatography (HPTLC) analyses were performed to investigate the chemical intactness of the substrate at the end of the uptake studies.

**Fig. 6:**
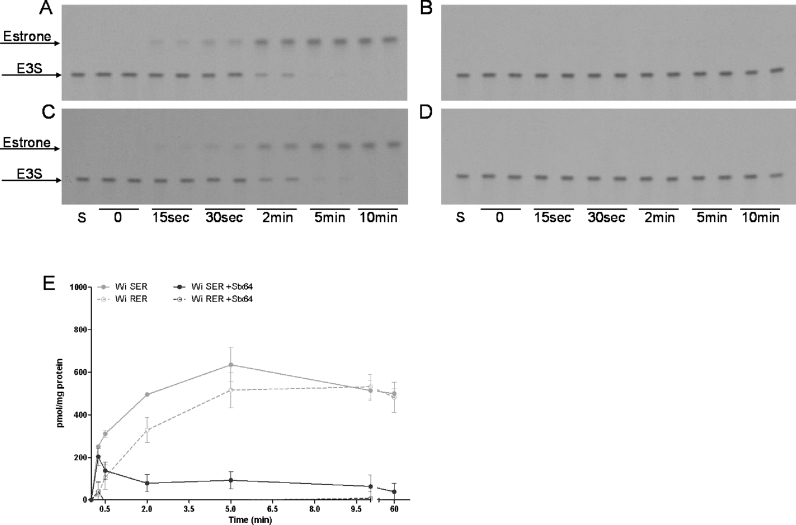
Autoradiogram (A-D) and time-dependent uptake (E) of E3S in presence (B, D, E) and absence (A, C, E) of the STS inhibitor Stx64. **A-D:** Wi SER (A, B) and RER (C, D), either not preincubated (A, C) or preincubated with 1 μM Stx64 (B, D), were incubated for 0, 15 sec, 30 sec, 2 min, 5 min and 10 min with 5 μM E3S and processed as described in section 2.6. After chromatography, X-ray film was exposed for 1 month. S, incubation buffer containing 5 μM E3S as standard. **E:** Uptake of 5 μM E3S was determined in SER and RER fractions either without (light grey) or with (dark grey) Stx64 (1 μM) preincubation after different time points of incubation at 37 °C at an inward positive membrane potential as described in 2.5.1. Data are shown for a representative sample of 2 independent experiments. Uptake is calculated as mean pmol/mg protein ± SD from measurements at least in triplicates.

HPTLC analysis showed time-dependent E3S hydrolysis in SER (Fig. 6A) as well as in RER (Fig. 6C) vesicles. At 15 sec of incubation 92% and 94% of the substrate was still intact in RER and SER vesicles, respectively. Only 18% and 42% of E3S was not hydrolyzed after 2 min in SER and RER vesicles, respectively, indicating slightly slower E3S hydrolysis in RER as compared to SER. Complete degradation in both ER fractions was observed after 10 min. Hence, the binding of the estrone, which carries the radioactive label may indeed be partitioning into the membrane of the vesicles used for the transport studies. As the catalytic site of steroidsulfatase (STS) is located within the ER lumen, vesicles were preincubated with the irreversible STS inhibitor Stx64 for 30 min at room temperature followed by 30 min incubation on ice prior to HPTLC analyses. Indeed, E3S was prevented from hydrolysis at any time point of incubation in SER and RER (Fig. 6B and D).

The effect of STS inhibition on E3S uptake was therefore also studied in SER and RER vesicles either pretreated or not pretreated with Stx64. Similar to the time-dependent hydrolysis, E3S uptake decreased with time in Stx64 treated vesicles (Fig. 6E) reaching a maximal inhibitory effect after 5 min of incubation.

### 3.5 Inhibition studies

To compare functional characteristics of TC, E3S and E17βG transport in more detail, inhibition studies were performed. Furthermore, a possible involvement of already identified ER transporters, including the glucose-6-phosphate transporter [37–39] and the UDPGA transporter [40–42], in TC, E3S, and E17βG transport was investigated. Therefore, UDPGA and glucose-6-phosphate (G6P) were included in inhibition experiments. In case of inhibition studies with G6P, vanadate treatment of vesicles was included to inhibit the glucose-6-phosphatase [43]. A control experiment without vanadate treated vesicles was also performed.

#### 3.5.1 Cis- inhibition studies on TC transport

As shown in Table 3, TC transport was inhibited by both estrogens, E3S and E17βG, at an incubation time of 30 sec. E17βG significantly decreased TC uptake in SER and RER vesicles to 53% and 71% of the control respectively. E3S also showed a significant inhibitory effect on the uptake of TC, reducing uptake rates to 60% of the control in SER vesicles. Although TC transport was also reduced to 71% of the control in RER vesicles, inhibition did not reach statistical significance.

As Stx64 was shown to inhibit E3S uptake, its effect on TC uptake was also studied, but no effect could be observed. Likewise, neither UDPGA nor G6P (with or without vanadate treatment) affected TC uptake in SER and RER vesicles.

#### 3.5.2 Cis- inhibition studies on E3S transport

Table 4 shows the results of inhibition studies for E3S uptake at an incubation time of 15 sec. E3S uptake was significantly inhibited by E17βG in SER, showing a decrease to 73% of the control. Although the E3S uptake into RER was reduced to 62% in presence of E17βG, this inhibitory effect was not statistically significant. TC did not affect E3S uptake, neither in SER nor in RER fractions.

E3S uptake was also significantly inhibited in vanadate treated vesicles to 54-57% of control in SER. In RER the inhibitory effect was even more pronounced and E3S uptake was almost completely inhibited. The irreversible STS inhibitor Stx64 decreased E3S transport to 80% and 76% in SER and RER fractions respectively, but did not reach statistical significance. No inhibitory effect was observed for UDPGA and G6P.

#### 3.5.3 Trans- inhibition studies on E17βG efflux

For E17βG efflux, trans-inhibition studies were performed at an incubation time of 5 sec. As shown in Table 5, E3S significantly inhibited E17βG efflux out of SER and RER vesicles. Compared to controls, E17βG efflux was inhibited by 59% in SER. In RER the inhibitory effect reduced E17βG efflux to 66% compared to the control, which did not reach statistical significance.

Neither TC, nor one of the other tested compounds, including UDPGA, Stx64 or G6P (with or without vanadate) affected E17βG efflux.

## 4 Discussion

The extensive network of the ER accounts for more than 50% of the total membrane surface and about 15% of the total volume in hepatocytes [44] and residents enzymes involved in biotransformation reactions, including UGTs [16] and the STS [10–12]. As both enzymes [12,19] have their catalytic sites within the ER lumen, their substrates, co-substrates as well as end products have to cross the ER membrane. Based on their physicochemical properties these compounds are not favored for passive diffusion and necessitate transport systems.

Therefore we investigated the transport of the STS model substrate E3S and the typical UGT generated E17βG across the membrane of the smooth (SER) and the rough (RER) ER subcompartment isolated from rat liver. Successful separation of SER and RER vesicles isolated as well as only marginal contamination of SER and RER with plasma membrane was shown by Western blot analysis. Electrogenic TC uptake, shown in the present study, is in accordance to previous data [25] and therefore confirms the functionality of the vesicles used in our study. In addition, we demonstrated bidirectional TC transport across SER and RER, with higher uptake rates in SER compared to RER. For E17βG, a fast efflux out of SER and RER vesicles was shown, while no clear protein-mediated uptake was observed for this compound. Contrary, E3S uptake increased with time in SER and RER vesicles. Likewise similar time-dependency was observed in STS mediated E3S hydrolysis. For all three studied substrates, uptake characteristics only minimally differ between wild-type and the mutant TR^−^ rats, which lack of the efflux pump Mrp2.

Electrogenic TC uptake with higher uptake rates at an intravesicular positive K^+^ diffusion potential has previously been reported in total microsomes, in ER enriched microsomes [25] as well as in SER vesicles [45] isolated from rat liver. Hence, we used in the present study TC transport as a positive control. TC transport into SER has been suggested to be accomplished by the microsomal enzyme epoxide hydrolase based on experiments with immunoprecipitation and reconstitution in proteoliposomes [45]. However, no differences in bile salt uptake into wild-type BHK-21 cells and rat epoxide hydrolase transfected BHK-21 cells were later found, indicating that this enzyme is unlikely responsible for TC transport across the plasma membrane [46] and consequently also not into SER. In rodents, TC uptake into hepatocytes is mediated by Oatp1a1, Oatp1a4, and Oatp1b2 located at the basolateral membrane [47]. Oatp1a1 and Oatp1a4 expressing *Xenopus laevis* oocytes showed bidirectional TC transport [48]. Additionally, TC transport via Oatp1a1 was not affected by a K^+^ diffusion membrane potential in *Xenopus laevis* oocytes [49]. Contrary, our observations showed higher TC transport rates into SER and RER vesicles at an inwardly directed K^+^ gradient. Besides Oatps, the major part of TC uptake in hepatocytes is mediated by the Na^+^- taurocholate co-transporting polypeptide (Ntcp) in a sodium-dependent manner [50]. However, in our study sodium dependent uptake of TC accounted for only 20-30% of total TC uptake, which is much less than in isolated basolateral plasma membrane vesicles [51]. Based on TC transport characteristic shown in our study and a lack of significant amounts of Oatp1a1 and Ntcp protein in our SER and RER preparations, these TC transporters seem not to be involved in TC transport across SER and RER membranes. At present, the physiologic role of possible taurocholate transport into the ER is not fully understood. While glucuronidation of bile acids has been reported to be mediated by UGT1A3 [52], this pathway is under most situations minor in hepatocellular bile salt homeostasis [53]. Under prolonged cholestasis, glucuronidation of bile acids may act as an alternative biotransformation pathway for ameliorating the cytotoxicity of bile acids [54]. Whether glucuronidated bile salts leave the lumen of the ER by the same transport systems as the one described here remains to be elucidated.

UGT mediated glucuronidation takes place within the ER lumen [19] and requires the exit of the reaction products back to the cytoplasm. This transport pathway has been investigated for different glucuronides, including 4-methylumbelliferyl-β-D-glucuronide [24], phenolphthalein-β-D-glucuronide [23], naphthol AS-BI glucuronide [23], morphine-3-glucuronide [23,55] and E17βG [22] using total rat liver microsomes. Glucuronide uptake increased with time in these studies and in accordance to our observations fast initial uptake rates were observed for E17βG [22], 4-methylumbelliferyl-β-D-glucuronide [24], phenolphthalein-β-D-glucuronide and naphthol AS-BI glucuronide [23]. After the fast initial uptake phase, E17βG content was nearly constant over a time period of 20 sec to 1 min followed by a slow increase up to 1 h of incubation in our study. Such a biphasic uptake rate could be explained by a trans-inhibition of the transported substrate. Published data reported that a steady state level is reached after the initial uptake phase and was unchanged for at least 40 min or 60 min for E17βG [22] or phenolphthalein-β-D-glucuronide [23] respectively. This difference could be due to different isolation procedures of the membrane vesicles investigated. As we have shown here for E17βG in SER and RER, efflux of phenolphthalein-β-D-glucuronide [23], morphine-3-glucuronide [55] and E17βG [22] was also fast and time-dependent out of total rat liver microsomes. Comparing time-dependent uptake and efflux of different glucuronides, morphine-3-glucuronide [23,55] showed slower uptake and efflux rates as compared to 4-methylumbelliferyl-β-D-glucuronide [24], phenolphthalein-β-D-glucuronide [23] and E17βG [22], indicating the existence of different transport systems for the transport of glucuronides of different molecular weight and consequently size across the ER membrane [23]. An effect of molecular weight on glucuronide efflux has also been indicated for bilirubin glucuronide and 1-naphthol glucuronide in human liver microsomes [56]. To our knowledge the transport system(s) responsible for glucuronide transport across the ER membrane has not been identified at the molecular level so far. An antiport with the UGT co-substrate UDPGA has been postulated, as extravesicular UDPGA stimulated the efflux of p-nitrophenol- and phenolphthalein-glucuronide out of preloaded rat liver microsomes (trans-stimulation) [21]. UGTrel7 (SLC35D1) has been identified as UDPGA transporter [40–42] and therefore could be involved in the UDPGA/glucuronide antiport process. As we did not observe any effect of UDPGA on E17βG efflux in trans-inhibition studies, neither an antiport with UDPGA nor an involvement of the UDPGA transporter in E17βG efflux can be suggested from the present study. This is also in accordance to previous cis-inhibition studies showing that extravesicular UDPGA had no effect on the uptake of E17βG [22]. The clear and fast efflux of E17βG out of ER vesicles and the observed slow, biphasic uptake indicate an asymmetric transport system for this compound across the ER membrane, which preferentially export E17βG out of vesicles. As evidence is given for the existence of different transport systems for glucuronides of different molecular weights, substrate specificity of such an asymmetric efflux transporter has to be further evaluated.

After glucuronidation and translocation of formed glucuronides from the ER lumen back into the cytoplasm, glucuronides have to be excreted out of hepatocytes. At the canalicular membrane, the ATP binding cassette transporter Mrp2 is responsible for the ATP dependent export of glucuronides into the bile [5]. Recently, Mrp2 expression in the nuclear envelope, another subcompartment of the ER besides SER and RER, has been demonstrated by Western blotting in rat liver nuclear envelope fractions [57]. As we did not find an ATP dependent E17βG uptake in SER and RER vesicles, which was also previously absent in total liver microsomes [22], and Mrp2 expression was absent in our fractions an involvement of Mrp2 in E17βG transport across the ER membrane seems unlikely.

Compared to TC and E17βG, no data are available studying the transport of E3S across the ER membrane. Only one study investigated the transport of NaSO_4_, showing a bidirectional time-dependent but unsaturable transport in total microsomal vesicles isolated from rat liver [58]. However, E3S hydrolysis accomplished by STS within the ER lumen is the first step in estrogen synthesis [59] and hence requires E3S translocation from the cytoplasm into the ER lumen. Although STS is known to be expressed in placenta, STS activity and expression has also been detected in rat and human liver [17,60,61]. E3S is a known substrate of Oatps [47], which are therefore possible candidates for E3S transport across the ER membrane. However, only low expression of the basolateral rOatp1a1 was found in SER and RER vesicles used in our study, indicating that Oatps are not likely to be involved in E3S transport across in SER and RER vesicles. Based on its structure, STS itself has been discussed to be involved in E3S transport into the ER lumen. Two hydrophobic α-helices anchor the STS in the ER membrane and are postulated to form a tunnel which may allow the substrate to pass the ER membrane and get access to the catalytic site of the enzyme [12]. This suggestion is supported by our observations showing similarities in the time-dependency of E3S uptake and E3S hydrolysis. We demonstrated that in both, SER and RER fractions, E3S is hydrolyzed with time reaching complete E3S hydrolysis after 10 min. Furthermore, we demonstrated inhibition of E3S uptake in SER and RER vesicles by Stx64 and vanadate, which are both described to inhibit STS activity [62,63]. Indeed, we confirmed inhibition of STS mediated E3S hydrolysis by Stx64 using high performance thin layer chromatography. These results however do not completely rule out a tight functional coupling between STS and a yet unknown E3S transport system. An alternative explanation for the observed decrease in E3S hydrolysis in the presence of Stx64 could also be the reduced uptake of E3S due to presence of this STS inhibitor. Our current data do not allow to favor either of these possibilities.

**Fig. 7:**
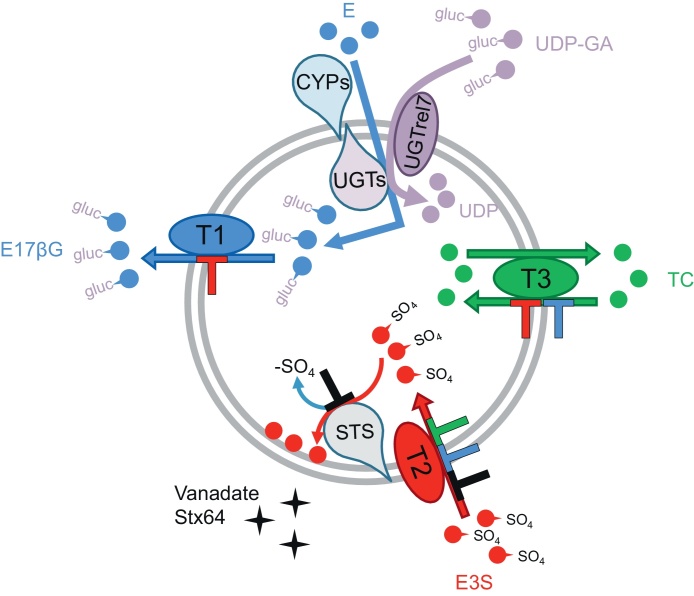
Schematic overview of postulated transport systems (T1, T2, T3) for E17βG, E3S, and TC in ER vesicles. Transport and inhibitory effect (⊥) of the different compounds are indicated by the different colors used. E, estradiol; E17βG, estradiol-17β-glucuronide; E3S, estrone-3-sulfate; TC, taurocholate; UDP-GA, UDP-glucuronic acid; CYPs, cytochrome P450s; UGTs, UDP-glucuronosyltransferases, STS, steroidsulfatase.

In conclusion, our study showed clear differences in the transport characteristics of TC, E3S, and E17βG across SER and RER membrane vesicles. Furthermore, the two STS inhibitors Stx64 and vanadate caused inhibition of E3S uptake but did not affect TC uptake or E17βG efflux. Therefore, we conclude that three different transport systems (as illustrated in Fig. 7) are likely involved in the transport of these compounds across the ER membrane. Our data also indicate that neither the known ER transporters UGTrel7 [40–42] and G6PT1 [37–39] nor the hepatocellular transporters Ntcp, Oatp1a1 and Mrp2 seem to be involved in the translocation of TC, E3S, and E17βG across the ER membrane. Based on our Western blot data, we conclude that the latter transporters are only found at minimal levels and are therefore unlikely relevant for metabolite transport processes in the ER.

## Figures and Tables

**Table 1:** Cyp3a2 and Sec61α expression normalized to PDI expression.

Rat strain	Fraction	Cyp3a2/PDI ratio	Sec61α/PDI ratio
Wistar	SER	1.60	1.99
	RER	0.86	0
TR^−^	SER	0.38	2.49
	RER	0.023	0

Data shown in the table represent the mean ratio of the areas measured by densitometry for Cyp3a2, Sec61α and PDI expression of two independent vesicle preparations, including the one shown in Fig. 1.

**Table 2:** E17βG uptake in absence and presence of ATP in Wistar SER and RER fractions.

Incubation time	SER			RER		
	-ATP	+ ATP		-ATP	+ ATP	
	pmol/mg protein	pmol/mg protein	% of -ATP	pmol/mg protein	pmol/mg protein	% of -ATP
15sec	129 ± 7	116 ± 22	90 ± 17	136 ± 9	108 ± 9	80 ± 6
1 min	138 ± 17	159 ± 18	115 ± 13	132 ± 5	135 ± 8	102 ± 6
5 min	181 ± 20	188 ± 13	104 ± 7	173 ± 14	161 ± 14	93 ± 8
2 h	270 ± 20	265 ± 8	98 ± 3	207 ± 11	223 ± 11	108 ± 5

Data of E17βG uptake in absence and presence of ATP at the given time points of incubation are shown for a representative sample of 2 independent experiments using vesicles from different vesicle preparations. Data are given as mean pmol/mg protein ± SD from measurements at least in triplicates.

**Table 3:** Summary of cis-inhibition studies of TC uptake at an incubation time of 30 sec.

Inhibitor (conc.)	SER			RER		
	Control	+ Inhibitor		Control	+ Inhibitor	
	pmol/mg protein	pmol/mg protein	% of control	pmol/mg protein	pmol/mg protein	% of control
E17βG (100 μM)	**37** **±** **2**	**20** **±** 1***	**53** **±** **3**	**18** **±** **3**	**13** **±** 2*	**71** **±** **3**
E3S (100 μM)	**33** **±** **5**	**20** **±** 4*	**60** **±** **5**	17 ± 4	12 ± 3	71 ± 5
UDPGA (4 mM)	38 ± 2	36 ± 5	94 ± 8	17 ± 3	15 ± 3	88 ± 3
Stx64 (1 μM)	38 ± 3	34 ± 5	90 ± 7	18 ± 2	17 ± 4	94 ± 12
G6P (1 mM)	36 ± 3	32 ± 1	90 ± 5	23 ± 5	21 ± 5	88 ± 7
Vanadate (1 mM)	36 ± 3	33 ± 1	93 ± 5	23 ± 5	21 ± 4	92 ± 5
Vanadate (1 mM) G6P (1 mM)	36 ± 3	32 ± 2	90 ± 5	23 ± 5	20 ± 4	87 ± 9

Data of TC uptake in absence (Control) and presence (+ Inhibitors) of the given inhibitors are given as mean pmol/mg protein ± SD of at least 3 independent experiments using vesicles from different vesicle preparations. Statistically significant difference in uptake rates compared to the control is given in bold. P-values are indicated by asterisks as followed: * for p < 0.05 and *** for p < 0.001.

**Table 4:** Summary of cis-inhibition studies of E3S uptake at an incubation time of 15 sec.

Inhibitor (conc.)	SER			RER		
	Control	+ Inhibitor		Control	+ Inhibitor	
	pmol/mg protein	pmol/mg protein	% of control	pmol/mg protein	pmol/mg protein	% of control
E17βG (100 μM)	**303** **±** **52**	**220** **±** 43*	**73** **±** **7**	136 ± 73	90 ± 56	62 ± 10
TC (50 μM)	303 ± 52	261 ± 66	86 ± 12	136 ± 73	113 ± 58	95 ± 41
UDPGA (4 mM)	281 ± 35	284 ± 41	101 ± 6	146 ± 17	156 ± 27	106 ± 6
Stx64 (1 μM)	319 ± 45	253 ± 26	80 ± 6	154 ± 23	120 ± 54	76 ± 26
G6P (1 mM)	259 ± 62	256 ± 69	99 ± 5	94 ± 43	92 ± 33	103 ± 18
Vanadate (1 mM)	**259** **±** **62**	**129** **±** 29**	**54** **±** **25**	**94** **±** **43**	**4** **±** 6*	**4** **±** **4**
Vanadate (1 mM) G6P (1 mM)	**259** **±** **62**	**137** **±** 27**	**57** **±** **26**	**94** **±** **43**	**11** **±** 14*	**12** **±** **11**

Data of E3S transport in absence (Control) and presence (+ Inhibitors) of the given inhibitors are given as mean pmol/mg protein ± SD of at least 3 independent experiments using vesicles from different vesicle preparations. Statistically significant difference in uptake rates compared to the control is given in bold. P-values are indicated by asterisks as followed: * for p < 0.05; and ** for p < 0.01.

**Table 5:** Summary of trans-inhibition studies of E17βG efflux at an incubation time of 5 sec.

Inhibitor (conc.)	SER			RER		
	Control	+ Inhibitor		Control	+ Inhibitor	
	pmol/mg protein/5sec	pmol/mg protein/5sec	% of control	pmol/mg protein/5sec	pmol/mg protein/5sec	% of control
E3S (100 μM)	**22** **±** **2**	**9** **±** 5*	**41** **±** **19**	19 ± 4	13 ± 2	66 ± 6
TC (50 μM)	19 ± 2	21 ± 5	108 ± 16	18 ± 3	21 ± 6	115 ± 18
UDPGA (4 mM)	18 ± 1	20 ± 3	111 ± 20	18 ± 4	19 ± 6	103 ± 14
Stx64 (1 μM)	21 ± 1	19 ± 3	91 ± 16	21 ± 5	20 ± 5	96 ± 9
G6P (1 mM)	20 ± 4	20 ± 5	99 ± 11	20 ± 1	21 ± 2	105 ± 3
Vanadate (1 mM)	20 ± 4	24 ± 3	118 ± 11	20 ± 1	20 ± 3	105 ± 14
Vanadate (1 mM) G6P (1 mM)	20 ± 4	24 ± 3	118 ± 11	20 ± 1	21 ± 5	104 ± 16

Data of E17βG efflux in absence (Control) and presence (+ Inhibitors) of the given inhibitors are given as mean pmol/mg protein/5 sec transported out of the vesicles ± SD of at least 3 independent experiments using vesicles from different vesicle preparations. Statistically significant difference in efflux rates compared to the control is given in bold. P-values are indicated by asterisks as followed: * for p < 0.05.

## References

[1] Vavricka S.R., Van Montfoort J., Ha H.R., Meier P.J., Fattinger K. (2002). Interactions of rifamycin SV and rifampicin with organic anion uptake systems of human liver. Hepatology.

[2] Zamek-Gliszczynski M.J., Hoffmaster K.A., Nezasa K., Tallman M.N., Brouwer K.L. (2006). Integration of hepatic drug transporters and phase II metabolizing enzymes: mechanisms of hepatic excretion of sulfate, glucuronide, and glutathione metabolites. Eur J Pharm Sci.

[3] Rowland A., Miners J.O., Mackenzie P.I. (2013). The UDP-glucuronosyltransferases: their role in drug metabolism and detoxification. Int J Biochem Cell Biol.

[4] Zollner G., Trauner M. (2006). Molecular mechanisms of cholestasis. Wien Med Wochenschr.

[5] Jedlitschky G., Leier I., Buchholz U., Hummel-Eisenbeiss J., Burchell B., Keppler D. (1997). ATP-dependent transport of bilirubin glucuronides by the multidrug resistance protein MRP1 and its hepatocyte canalicular isoform MRP2. Biochem J.

[6] Kartenbeck J., Leuschner U., Mayer R., Keppler D. (1996). Absence of the canalicular isoform of the MRP gene-encoded conjugate export pump from the hepatocytes in Dubin-Johnson syndrome. Hepatology.

[7] Jansen P.L., Peters W.H., Lamers W.H. (1985). Hereditary chronic conjugated hyperbilirubinemia in mutant rats caused by defective hepatic anion transport. Hepatology.

[8] Matsuura S., Fujii-Kuriyama Y., Tashiro Y. (1978). Immunoelectron microscope localization of cytochrome P-450 on microsomes and other membrane structures of rat hepatocytes. J Cell Biol.

[9] Radominska-Pandya A., Ouzzine M., Fournel-Gigleux S., Magdalou J. (2005). Structure of UDP-glucuronosyltransferases in membranes. Methods Enzymol.

[10] Dibbelt L., Herzog V., Kuss E. (1989). Human placental sterylsulfatase: immunocytochemical and biochemical localization. Biol Chem Hoppe Seyler.

[11] Kawano J., Aikawa E. (1987). Ultrastructural localization of arylsulfatase C activity in rat kidney. J Histochem Cytochem.

[12] Hernandez-Guzman F.G., Higashiyama T., Pangborn W., Osawa Y., Ghosh D. (2003). Structure of human estrone sulfatase suggests functional roles of membrane association. J Biol Chem.

[13] Voeltz G.K., Rolls M.M., Rapoport T.A. (2002). Structural organization of the endoplasmic reticulum. EMBO Rep.

[14] Porter K.R., Claude A., Fullam E.F. (1945). A Study of Tissue Culture Cells by Electron Microscopy: Methods and Preliminary Observations. J Exp Med.

[15] Ashley C.A., Peters T. (1969). Electron microscopic radioautographic detection of sites of protein synthesis and migration in liver. J Cell Biol.

[16] Chowdhury J.R., Novikoff P.M., Chowdhury N.R., Novikoff A.B. (1985). Distribution of UDPglucuronosyltransferase in rat tissue. Proc Natl Acad Sci U S A.

[17] Kawano J., Kotani T., Umeki K., Oinuma T., Ohtaki S., Aikawa E. (1989). A monoclonal antibody to rat liver arylsulfatase C and its application in immunohistochemistry. J Histochem Cytochem.

[18] Brown C.A., Black S.D. (1989). Membrane topology of mammalian cytochromes P-450 from liver endoplasmic reticulum. Determination by trypsinolysis of phenobarbital-treated microsomes. J Biol Chem.

[19] Vanstapel F., Blanckaert N. (1988). Topology and regulation of bilirubin UDP-glucuronyltransferase in sealed native microsomes from rat liver. Arch Biochem Biophys.

[20] Bossuyt X., Blanckaert N. (1994). Carrier-mediated transport of intact UDP-glucuronic acid into the lumen of endoplasmic-reticulum-derived vesicles from rat liver. Biochem J.

[21] Banhegyi G., Braun L., Marcolongo P., Csala M., Fulceri R., Mandl J. (1996). Evidence for an UDP-glucuronic acid/phenol glucuronide antiport in rat liver microsomal vesicles. Biochem J.

[22] Battaglia E., Gollan J. (2001). A unique multifunctional transporter translocates estradiol-17beta -glucuronide in rat liver microsomal vesicles. J Biol Chem.

[23] Csala M., Staines A.G., Banhegyi G., Mandl J., Coughtrie M.W., Burchell B. (2004). Evidence for multiple glucuronide transporters in rat liver microsomes. Biochem Pharmacol.

[24] Revesz K., Tutto A., Margittai E., Banhegyi G., Magyar J.E., Mandl J. (2007). Glucuronide transport across the endoplasmic reticulum membrane is inhibited by epigallocatechin gallate and other green tea polyphenols. Int J Biochem Cell Biol.

[25] Kast C., Stieger B., Winterhalter K.H., Meier P.J. (1994). Hepatocellular transport of bile acids. Evidence for distinct subcellular localizations of electrogenic and ATP-dependent taurocholate transport in rat hepatocytes. J Biol Chem.

[26] Meier P.J., Sztul E.S., Reuben A., Boyer J.L. (1984). Structural and functional polarity of canalicular and basolateral plasma membrane vesicles isolated in high yield from rat liver. J Cell Biol.

[27] Lowry O.H., Rosebrough N.J., Farr A.L., Randall R.J. (1951). Protein measurement with the Folin phenol reagent. J Biol Chem.

[28] Meier P.J., Spycher M.A., Meyer U.A. (1981). Isolation and characterization of rough endoplasmic reticulum associated with mitochondria from normal rat liver. Biochim Biophys Acta.

[29] Eckhardt U., Schroeder A., Stieger B., Hochli M., Landmann L., Tynes R. (1999). Polyspecific substrate uptake by the hepatic organic anion transporter Oatp1 in stably transfected CHO cells. Am J Physiol.

[30] Stieger B., Hagenbuch B., Landmann L., Hochli M., Schroeder A., Meier P.J. (1994). In situ localization of the hepatocytic Na+/Taurocholate cotransporting polypeptide in rat liver. Gastroenterology.

[31] Madon J., Hagenbuch B., Landmann L., Meier P.J., Stieger B. (2000). Transport function and hepatocellular localization of mrp6 in rat liver. Mol Pharmacol.

[32] Boyer J.L., Meier P.J. (1990). Characterizing mechanisms of hepatic bile acid transport utilizing isolated membrane vesicles. Methods Enzymol.

[33] Adelman M.R., Blobel G., Sabatini D.D. (1974). Nondestructive separation of rat liver rough microsomes into ribosomal and membranous components. Methods Enzymol.

[34] Kessler M., Acuto O., Storelli C., Murer H., Muller M., Semenza G. (1978). A modified procedure for the rapid preparation of efficiently transporting vesicles from small intestinal brush border membranes. Their use in investigating some properties of D-glucose and choline transport systems. Biochim Biophys Acta.

[35] Gilchrist A., Au C.E., Hiding J., Bell A.W., Fernandez-Rodriguez J., Lesimple S. (2006). Quantitative proteomics analysis of the secretory pathway. Cell.

[36] Jager W., Sartori M., Herzog W., Thalhammer T. (1998). Genistein metabolism in liver microsomes of Wistar and mutant TR(-)-rats. Res Commun Mol Pathol Pharmacol.

[37] Gerin I., Veiga-da-Cunha M., Achouri Y., Collet J.F., Van Schaftingen E. (1997). Sequence of a putative glucose 6-phosphate translocase, mutated in glycogen storage disease type Ib. FEBS Lett.

[38] Marcolongo P., Fulceri R., Giunti R., Margittai E., Banhegyi G., Benedetti A. (2012). The glucose-6-phosphate transport is not mediated by a glucose-6-phosphate/phosphate exchange in liver microsomes. FEBS Lett.

[39] Senesi S., Marcolongo P., Kardon T., Bucci G., Sukhodub A., Burchell A. (2005). Immunodetection of the expression of microsomal proteins encoded by the glucose 6-phosphate transporter gene. Biochem J.

[40] Hiraoka S., Furuichi T., Nishimura G., Shibata S., Yanagishita M., Rimoin D.L. (2007). Nucleotide-sugar transporter SLC35D1 is critical to chondroitin sulfate synthesis in cartilage and skeletal development in mouse and human. Nat Med.

[41] Kobayashi T., Sleeman J.E., Coughtrie M.W., Burchell B. (2006). Molecular and functional characterization of microsomal UDP-glucuronic acid uptake by members of the nucleotide sugar transporter (NST) family. Biochem J.

[42] Muraoka M., Kawakita M., Ishida N. (2001). Molecular characterization of human UDP-glucuronic acid/UDP-N-acetylgalactosamine transporter, a novel nucleotide sugar transporter with dual substrate specificity. FEBS Lett.

[43] Singh J., Nordlie R.C., Jorgenson R.A. (1981). Vanadate: a potent inhibitor of multifunctional glucose-6-phosphatase. Biochim Biophys Acta.

[44] Weibel E.R., Staubli W., Gnagi H.R., Hess F.A. (1969). Correlated morphometric and biochemical studies on the liver cell. I. Morphometric model, stereologic methods, and normal morphometric data for rat liver. J Cell Biol.

[45] Alves C., von Dippe P., Amoui M., Levy D. (1993). Bile acid transport into hepatocyte smooth endoplasmic reticulum vesicles is mediated by microsomal epoxide hydrolase, a membrane protein exhibiting two distinct topological orientations. J Biol Chem.

[46] Honscha W., Platte H.D., Oesch F., Friedberg T. (1995). Relationship between the microsomal epoxide hydrolase and the hepatocellular transport of bile acids and xenobiotics. Biochem J.

[47] Hagenbuch B., Meier P.J. (2004). Organic anion transporting polypeptides of the OATP/SLC21 family: phylogenetic classification as OATP/SLCO superfamily, new nomenclature and molecular/functional properties. Pflugers Arch.

[48] Li L., Meier P.J., Ballatori N. (2000). Oatp2 mediates bidirectional organic solute transport: a role for intracellular glutathione. Mol Pharmacol.

[49] Martinez-Becerra P., Briz O., Romero M.R., Macias R.I., Perez M.J., Sancho-Mateo C. (2011). Further characterization of the electrogenicity and pH sensitivity of the human organic anion-transporting polypeptides OATP1B1 and OATP1B3. Mol Pharmacol.

[50] Stieger B. (2011). The role of the sodium-taurocholate cotransporting polypeptide (NTCP) and of the bile salt export pump (BSEP) in physiology and pathophysiology of bile formation. Handb Exp Pharmacol.

[51] Zimmerli B., Valantinas J., Meier P.J. (1989). Multispecificity of Na + -dependent taurocholate uptake in basolateral (sinusoidal) rat liver plasma membrane vesicles. J Pharmacol Exp Ther.

[52] Trottier J., Verreault M., Grepper S., Monte D., Belanger J., Kaeding J. (2006). Human UDP-glucuronosyltransferase (UGT)1A3 enzyme conjugates chenodeoxycholic acid in the liver. Hepatology.

[53] Hofmann A.F. (2007). Why bile acid glucuronidation is a minor pathway for conjugation of endogenous bile acids in man. Hepatology.

[54] Frohling W., Stiehl A. (1976). Bile salt glucuronides: identification and quantitative analysis in the urine of patients with cholestasis. Eur J Clin Invest.

[55] Revesz K., Toth B., Staines A.G., Coughtrie M.W., Mandl J., Csala M. (2013). Luminal accumulation of newly synthesized morphine-3-glucuronide in rat liver microsomal vesicles. Biofactors.

[56] Waddell I.D., Robertson K., Burchell A., Hume R., Burchell B. (1995). Evidence for glucuronide (small molecule) sorting by human hepatic endoplasmic reticulum. Mol Membr Biol.

[57] Rosales R., Monte M.J., Blazquez A.G., Briz O., Marin J.J. (2012). ABCC2 is involved in the hepatocyte perinuclear barrier for small organic compounds. Biochem Pharmacol.

[58] Csala M., Senesi S., Banhegyi G., Mandl J., Benedetti A. (2005). Characterization of sulfate transport in the hepatic endoplasmic reticulum. Arch Biochem Biophys.

[59] Ghosh D. (2007). Human sulfatases: a structural perspective to catalysis. Cell Mol Life Sci.

[60] Dolly J.O., Dodgson K.S., Rose F.A. (1972). Studies on the oestrogen sulphatase and arylsulphatase C activities of rat liver. Biochem J.

[61] Selcer K.W., Difrancesca H.M., Chandra A.B., Li P.K. (2007). Immunohistochemical analysis of steroid sulfatase in human tissues. J Steroid Biochem Mol Biol.

[62] Dibbelt L., Kuss E. (1991). Human placental sterylsulfatase. Interaction of the isolated enzyme with substrates, products, transition-state analogues, amino-acid modifiers and anion transport inhibitors. Biol Chem Hoppe Seyler.

[63] Woo L.L., Purohit A., Malini B., Reed M.J., Potter B.V. (2000). Potent active site-directed inhibition of steroid sulphatase by tricyclic coumarin-based sulphamates. Chem Biol.

